# Mapping student engagement in health professions education policy and decision-making: a scoping review

**DOI:** 10.1186/s12909-024-05283-8

**Published:** 2024-03-22

**Authors:** Hanieh Neshastesaz Kashi, Salime Goharinezhad, Samira Soleimanpour, Ali Haji Mohammadi

**Affiliations:** 1 Centre for Educational Research in Medical Sciences (CERMS), Faculty of Medicine, Iran University of Medical Sciences, Tehran, Iran; 2 School of Health, Wellbeing and Social Care, Faculty of Wellbeing, Education and Language Studies, The Open University, Walton Hall, Kents Hill, Milton Keynes, MK7 6AA, UK, England; 3 Education Development Center (EDC), Iran University of Medical Sciences, Tehran, Iran

**Keywords:** Health professions education, Medical education, Student engagement, Policy-making, Decision-making, Scoping review

## Abstract

**Background:**

“Student engagement” (SE) is gaining momentum as an approach to improve the performance of health professions education (HPE). Nevertheless, despite the broad studies about the role of students in various areas, little is known about the role of SE in policy and decision-making activities. This study aimed to map SE in policy and decision-making regarding terms and definitions, engagement models, influencing factors, outcomes and achievements, and the interconnection between the influencing factors.

**Method:**

Five databases (PubMed, Scopus, ProQuest, Web of Science, and ERIC) were systematically searched from Jan 1, 1990, to Nov 12, 2022. The review was followed according to the Arksey and O’Malley framework for scoping reviews and reported according to the PRISMA-ScR guidelines. We included articles published in English focusing on HPE policy and decision-making. The authors summarized and synthesized the findings into themes, subthemes, tables, and models.

**Results:**

Of the 22 articles included in the full-text review, terms and definitions were tabled, and three themes were extracted: 1. models of SE, in which 10 studies (45.5%) presented the highly structured formal models as Organizations, 5 studies (22.7%) reported less-structured community and group as Programs, and 7 studies (31.8%) engaged students only in surveys or interviews as Perspective; 2. Factors influencing SE, that were categorized into 7 subthemes: structural, environmental, and motivational factors, member characteristics, training and mentoring, member relationships, valuing and recognizing. 3. Outcomes and achievements of SE related to systems and members. The interconnection between influencing factors is also demonstrated as a conceptual model.

**Discussion:**

There are various SE models in HPE policy and decision-making, which are mapped and categorized depending on the degree of formality, structuredness, and level of engagement. In our study, three more common SE models in HPE policy and decision-making were investigated. Additionally, these collaborative methods emphasized curriculum development and quality assurance and employed students in these activities. It is worth mentioning that to make SE models more efficient and sustainable, several influencing factors and their interconnections should be considered.

**Supplementary Information:**

The online version contains supplementary material available at 10.1186/s12909-024-05283-8.

## Introduction

Recent research indicates that health profession education (HPE) needs to undergo significant changes in engaging students [1–3]. This sense of change is affected by new trends such as progression in educational theories and student-centred approaches [4]. To achieve this, health sciences universities must adopt a ‘student as partner’ model instead of treating students as passive consumers. This requires a cultural shift and changes in systems and processes [3, 5, 6]. Students should be recognized as key stakeholders in the design and implementation of educational programs, and their perspectives and ideas should be actively solicited [7, 8]. Educational institutions that give precedence to continuous improvement have prioritized “Student Engagement” (SE) as an essential element of their organizational strategies [9, 10]. Thus, universities should develop new traditions that provide opportunities for SE to improve their institutional effectiveness [3].

As Peters [11] and Trowler [12] discuss, “Student Engagement encompasses a wide range of collaborative activities with staff in universities, which enhance student learning and development and contribute to improving the quality of academic environment and culture in the institutes.” In a more consolidated way, different stages of SE are distinguished as a continuum with *consultation* being the least engaged and *involvement*, *participation*, and *student-staff partnership* becoming progressively more engaged [13, 14]. *Consultation* entails simply sharing opinions, while *involvement* allows for a more active role. *Participation* is a more formal and structured collaboration with staff, and *student-staff partnership* involves shared ownership in decision-making for both processes and outcomes [3, 14].

Considering the broad concept and various dimensions of SE and its impact on creating novel educational changes, the key point for constructing a more efficient educational system is to use students’ power for political decision-making [15]. Additionally, students’ participation in university governance can facilitate a productive relationship between students, faculty, and administrators, and by working together, institutions can remain aware of how students view their function. Furthermore, program directors, coordinators, and students achieve a greater awareness that promotes student-staff collaboration and may benefit both the education program and the stakeholders [3]. In fact, university officials should delegate their power to students to control their education and development, making them feel valued [3, 16]. Based on the literature, administrative committees with student members are more concerned with students’ issues. Moreover, involvement with organizations can enhance students’ viewpoint toward the workplace and their future careers [15]. During these engagements, students develop practical competencies that would be beneficial for their profession, such as strategic thinking, debating, networking, efficient communication, and organizing [3, 17].

According to these findings, we decided to investigate the categories of SE among health sciences universities in all types of policy and decision-making activities inspired by the ASPIRE Student Engagement Criteria [18]^1^. The classification applied in this guideline allows us to better understand the areas of SE, especially the critical role of health profession students in university governance and policy-making bodies [19]. The ASPIRE initiative has been accepted by scholars and the academic community since 2012, and it has encouraged health sciences universities to plan for achieving excellence in SE as a portion of their mission [20].

However, we investigated research and found papers that discussed the theoretical perspectives and frameworks of SE. Also, many studies have examined the role of SE in teaching, learning or evaluations and have mostly studied medical students. There is a lack of research studying the students as partners and presenting the practical approaches to engaging students, especially in HPE policy and decision-making. Thus, we decided to capture all health professions students in our study and explore the published studies on SE in HPE policy and decision-making among health sciences universities. In doing so, we used the ASPIRE criteria to identify various types of operational models for involving students in governance and policy-making.

The primary aim of this study is to comprehensively investigate the various models/frameworks of SE in HPE policy and decision-making within universities. Additionally, the study seeks to explore the outcomes and achievements resulting from the implementation of SE in HPE policy and decision-making. Furthermore, the study aims to identify and analyze the factors that influence SE in HPE policy and decision-making, and to examine the interconnections among these influencing factors. By delving into these aspects, the study aims to provide a comprehensive understanding of SE in HPE and provide insights that can inform effective policy-making and decision-making processes in universities [3, 21, 22].

## Method

The review was followed according to the Arksey and O’Malley [23] framework in the form of a 5-step methodology as well as Joanna Briggs Institute (JBI) methodology [24]. In addition, we utilized the Preferred Reporting Items for Systematic Reviews and Meta-Analyses extension for Scoping Reviews (PRISMA-ScR) as a guide for reporting our findings.

### Identification of the research question

This review is guided by the following research questions:

- What are terms used in the literature referring to SE in HPE policy and decision-making and their definitions?
- What are the models of SE in HPE policy and decision-making in universities?
- What factors influence SE in HPE policy and decision-making in universities?
- What are the outcomes and achievements of the SE in HPE policy and decision-making in universities?
- How influencing factors of SE in HPE policy and decision-making in universities are interconnected?

### Identifying relevant studies and search strategy

An electronic search was launched through the following databases for literature published in PubMed, Scopus, ProQuest, Web of Science, and Education Resources Information Centre (ERIC). The search strategies for all of these databases were shaped with the help of an academic librarian (the fourth author S. S.) using keywords extracted from the study questions and aims. Some of the terms used for searching are provided: (“student engagement” OR “student partnership” OR “learner engagement” OR “student involvement” OR “student participation” OR “student contribution” AND (govern* OR “policy making” OR “decision making” OR “management”). Appendix S1 provides a detailed search strategy. Following the databases, we identified additional articles by searching the references list in key peer-reviewed articles related to the subject of SE. Moreover, we included several published articles by hand-searching in key medical education journals (see Appendix S2).

### Eligibility criteria for inclusion of publication

According to our inclusion criteria, we selected papers with the following characteristics: 1. peer-reviewed articles published or on-press in English; 2. being part of the undergraduate HPE disciplines (medicine, pharmacy, dentistry, veterinary, nursing, and public health disciplines); 3. the focus of research is on SE in HPE policy and decision-making activities in universities, such as SE models, their outcomes, and influencing factors; and 4. The study is done in health sciences faculties. The following studies were excluded: 1. non-English papers, 2. articles with no existing full texts, 3. studies involving students merely in clinical decision-making, teaching and learning policies and just in curriculum evaluation, and 4. conference abstracts, letters and editorials. Detailed inclusion and exclusion criteria are presented in Appendix S3.

We restricted the search to studies published from Jan 1, 1990, to Nov 12, 2022, whether published or on-press, without performing a quality assessment. The results were extracted by our fourth author (S.S.) based on the search strategy and then exported to EndNote 20.4.1 (Clarivate, U.S.A.). Two reviewers (H. N. and A. H.) independently screened the titles, abstracts and full papers at each level according to the inclusion and exclusion criteria, accompanied by consultations with the third reviewer (S. G.) in cases of non-agreement.

### Charting the data

Charting the data implemented via Google Sheets and the headings created based on JBI guideline [24] and through consensus of authors and were: title, first author, publication year, journal, study location, study design, aim of study, participants, study population size, data collection methods, main findings of study, term and definition related to SE, categories of SE, models of SE, influencing factors and prerequisites.

The study developed two categories of data charting in direct response to the research questions. The first category consists of models, frameworks, bodies, and forms of SE, while the second category includes influencing factors and prerequisites. To understand the outcomes and achievements of SE models, a separate category was not determined. Instead, titles such as “main findings of the study” and “aim of the study” were used. During the full-text review, we realized the great variety in how studies described the engagement of students. As a result, a separate column was created to extract different definitions of the terms related to SE.

To achieve a robust data extraction approach, the authors discussed and agreed on the mentioned key study characteristics that aligned with the research questions. As a pilot test, the authors independently extracted the data from the 10 random articles in Google Sheets to evaluate the efficiency and consistency of the sheet categories. After a meeting of discussion, the authors agreed on the refined format of the data extraction sheet. The remaining articles were allocated among the authors for data extraction based on the agreed sheet and key study characteristics.

### Collating, summarizing and reporting results

We started with deductive coding to identify main themes using the research questions and initial literature review, followed inductively through included studies to proceed with thematic analysis [25]. Firstly, the authors (H.N. and A.H.) collected a summary of each study regarding the above extraction criteria. Then, key findings of the summaries were detected and highlighted. In the next step, two authors sorted and categorized the key findings and ended up in sub-themes. One author (H.N.) synthesized the findings of the SE models and their outcomes and achievements, and the other author (A.H.) synthesized the influencing factors. Lastly, a relational content analysis approach (cognitive mapping) [26, 27] was also utilized by A.H. to demonstrate how influencing factors in SE are interconnected. The research team achieved consensus on a common set of themes as well as the conceptual model of influencing factors through iterative discussion. During the full-text review stage, we observed significant differences in how SE was defined or described across articles. As a result, we extracted the SE terms and definitions and compiled a table for comparison purposes.

## Results

### Study selection process

**Fig. 1:**
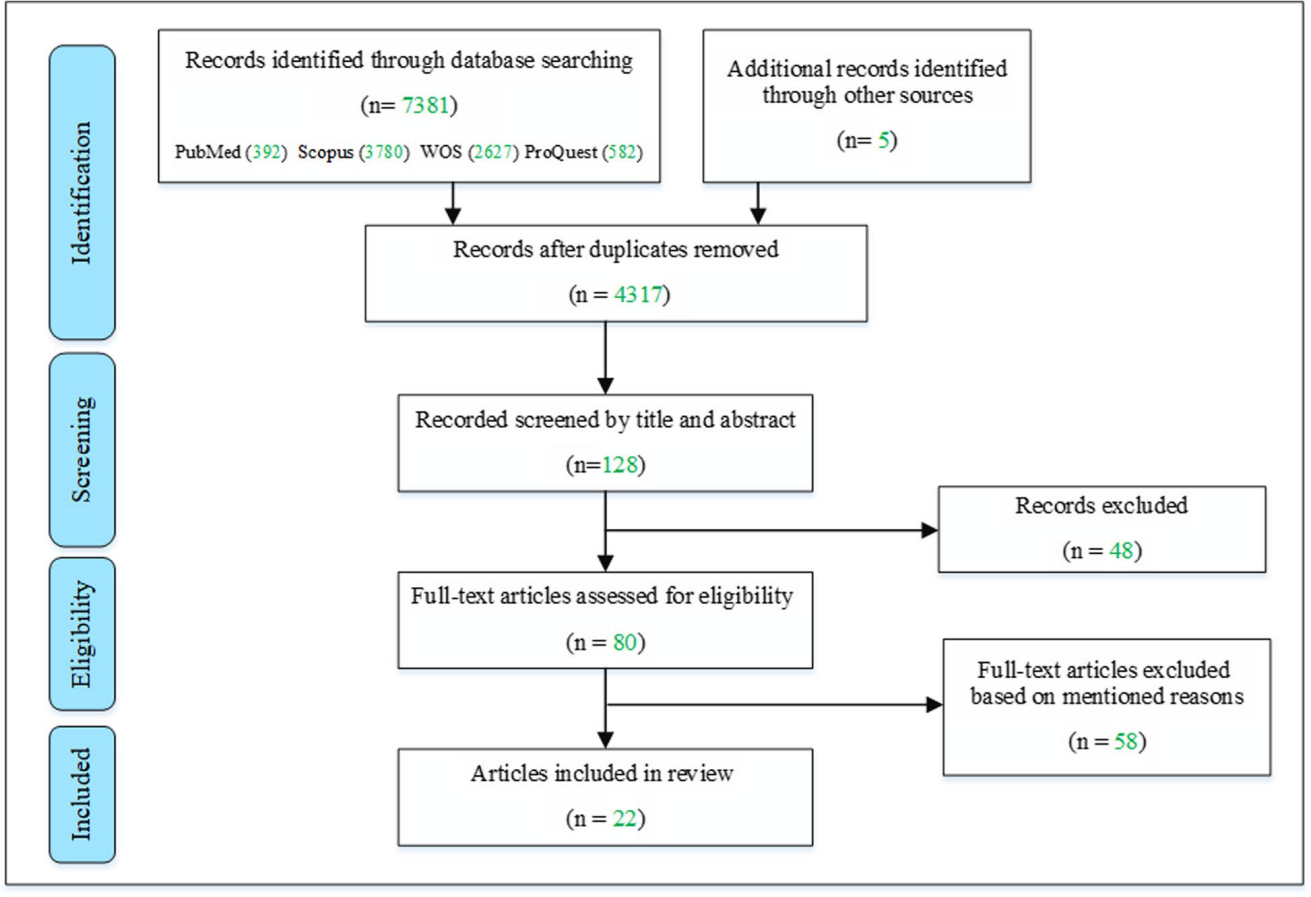
Flowchart indicating the study selection process, which is constructed in line with the PRISMA extension for scoping reviews guidelines [23]

Information about the different stages of the study selection process is illustrated in a PRISMA-ScR flowchart in Fig. 1. After the removal of 3069 duplicated articles, the title and abstract screening process included 128 articles according to our selection criteria. Eighty articles met the full-text eligibility criteria, and 48 were excluded because they did not have any accessible English full-text version or consider any models, programs, or frameworks related to SE in HPE policy and decision-making. Finally, 22 articles were included in this scoping review.

### Study characteristics

As shown in Table 1, studies from the USA were dominant (40.9%), with 59% of papers published in the last 6 years. Overall, the qualitative study design was the most common method (86.4%). However, the number of students involved in the studies was variable, and medical students had the predominant population size (68.2%) among the SE models. Moreover, 91% of papers targeted “curriculum development” or “quality assurance” as their categories utilizing student participation.

**Table 1:** Summary of the characteristics of the included studies (*N* = 22)

Publication year	1998–2004	*N* = 3 (13.6%)	2011–2016	*N* = 4 (18.1%)
	2005–2010	N = 2 (9%)	2017–2022	*N* = 13 (59%)
**Location**	USA	*N* = 9 (40.9%)	Belgium	N = 1 (4.5%)
	Netherland	N = 3 (13.6%)	Japan	N = 1 (4.5%)
	Australia	N = 3 (13.6%)	Slovenia	N = 1 (4.5%)
	Malaysia	N = 1 (4.5%)	Germany	N = 1 (4.5%)
	Canada	N = 1 (4.5%)	UK	N = 1 (4.5%)
**Method**	Qualitative	*N* = 19 (86.4%)	Mixed method	N = 3 (13.6%)
**Participants**	Medical students	*N* = 15 (68.2%)	Faculty, staff, and administrators	*N* = 8 (36.3%)
	Dentistry, pharmacy, and veterinary students			N = 2 (9%)
**Categories**	Curriculum development	*N* = 18 (81.8%)	Planning for the educational program	N = 3 (13.6%)
	Quality assurance	N = 4 (18.2%)	Faculty/staff development	N = 2 (9%)

### Terms and definitions of student engagement in health professions education policy and decision-making

Toward a deeper understanding of the SE concept, we have depicted the extended spectrum of the SE definitions used in various studies in Table 2. However, various terms have been used instead of “engagement” in studies, which are as follows: a) involvement, b) participation, c) cooperation, d) contribution, e) consultation f) coproduction, and g) partnership.

**Table 2:** Terms used in the literature referring to Student engagement and their definition

N.	Author/year	Term	Definition
**1**	Er, Hui Meng 2020	Student engagement in quality assurance	“Student engagement in quality assurance (QA) is extending from participation in course and learning environment evaluation, to involvement in structures and process at subject, faculty and institutional level. “ [28]
**2**	Galina Gheihman 2021	Curricular coproduction	“A process in which learners partner with educators to create their educational experiences, curricula, and learning environment. Learners may contribute to the design, implementation, and evaluation of both nonclinical and clinical curricula.” [29]
**3**	Meeuwissen, Stephanie N.E. 2019	Student engagement	“Increasing levels of student engagement are recognized and vary from consultation, involvement, and participation to partnership. **Student Consultation:** Students can express their perspectives **Student Involvement:** Students can take a more active role **Student participation:** An active role in a defined, collaborative process with staff **Student-staff partnership:** Joint ownership and decision-making over processes and outcomes.” [3]
**4**	Samantha E. Martens 2020	Student-staff partnership	“A collaborative, reciprocal process through which all participants have the opportunity to contribute equally, although not necessarily in the same ways, to curricular or pedagogical conceptualization, decision making, implementation, investigation or analysis.” [10]
**5**	Milles, Lennart Steffen 2019	Student engagement	“Represents a mutually beneficial collaborative approach between students and their institutions in higher education. It refers to a broad range of activities to enhance the learning and development of students, as well as the quality of the academic environment and institutional culture.” [30]
**6**	Patricio, Madalena 2016	Student engagement	“A psycho-social process, influenced by institutional and personal factors, and embedded within a wider social context, integrates the social-cultural perspective with the psychological and behavioral” [19]
**7**	Kassab, Salah Eldin 2022	Student engagement	**“Groccia’s multidimensional conceptual model:** In this model, student engagement is defined as the aspects of student academic experience in teaching, learning and research through interacting with other students, faculty and community at the cognitive (thinking), affective (feeling) and behavioral (doing) levels.” [5]

Regarding the research questions, we thematically analyzed the included studies. Accordingly, the findings are charted in Table 3 in which the main themes are 1) the models, 2) influencing factors, 3) and outcomes and achievements of SE in HPE policy and decision-making in universities. Each subtheme contains the recognized activities and/or the definition of that subtheme. At last, a conceptual model is also presenting how influencing factors interact.

**Table 3:** Three main themes, their subthemes, and activities or definitions extracted relating to SE in HPE policy and decision-making

Main themes	Subthemes	Activities or definitions		References
**1. Models of SE**	1. Organization	• Establish formal and well-structured models defined in the organizational chart to ensure long-term and sustainable SE. • Contribute to: ° Curriculum reform and co-direction (pre-clerkship and clerkship curriculum) ° Develop feedback and evaluation processes ° Expand innovative educational policies and guidelines • Examples of formal models include: ° Student Workgroup on Medical Education (SWME) ° Student Curricular Board (SCB) ° Partnered Educational Governance (PEG) ° Centre for Medical Education (CME) ° Student Module Co-directors ° Student Curriculum Review Team (SCRT)		[1, 3, 4, 8, 9, 30–34]
	2. Programs	• Utilize informal models or less structured committees and groups for short-term SE • Prepare management, leadership or advisory roles to embrace student-initiated policies for educational and institutional development • Examples of informal models include: ° Health Systems Science (HSS) ° Education Representatives program (Ed Reps)		[10, 35–38]
	3. Perspective	• Utilize cross-sectional surveys and interviews • Aim to incorporate student insights and opinions into curricular and staff development • Example: ° School of Medicine Resources Management System (SOMRMS)		[28, 29, 39–43]
**2. Influencing factors**	1. Structural factors	• Establish a well-structured and centralized SE organization • Encourage regular attendance at meetings • Ensure an appropriate balance of student power • Adopt a top-down management approach • Incorporate SE in curricular research projects, surveys, and need assessments. • Encourage students to independently organize themselves • Select new student members through an independent process led by the student committee • Promote clarity and transparency in defining and communicating roles and objectives • Continuously evaluate engagement models or programs		[1, 3, 4, 8–10, 28, 30, 31, 33, 37–39, 43]
	2. Environmental factors & institutional culture	• Foster a culture that prioritizes SE and values their opinions and feedback • Create a supportive environment that encourages them to express their views		[1, 10, 28, 37]
	3. Motivational Factors	• Consider internal sources such as: ° Personal growth ° Building competencies ° Extracurricular activities ° Community service ° Improving education	• Explore external sources including: ° Rewards ° Financial support ° Seed grants ° Final scores	[3, 10, 28, 30, 31, 36, 39, 41]
	4. Members characteristics	• Evaluate personal attributes including: ° Maturity ° Entrepreneurial spirit ° Responsibility ° Leadership ° Passion for education ° Self-confidence ° Diplomacy	° Open-mindedness ° Critical thinking ° Collaboration • Review candidates’ resumes and past SE activities • Seek faculty/staff members who value students’ opinions and are passionate about SE	[1, 3, 9, 28, 37–39]
	5. Training and mentoring	• Develop formal training programs for students and faculty/staff in the following areas: ° Constructive feedback skills ° Effective communication ° Leadership ° Familiarity with the HPE • Provide sufficient orientation about school policies and current issues • Facilitate mentoring of junior members by senior representatives • Ensure smooth transition of responsibilities and provide coaching to new representatives		[1, 3, 8–10, 28, 31, 34, 36, 38, 39, 43]
	6. Members relationship	• Work collaboratively based on the following: ° Reciprocal communication ° Equality ° Mutual respect ° Trust ° Open dialogues • Provide a non-threatening environment and safe space to share ideas • Ensure coordination among student representatives, consensus building, and adequate preparation for meetings		[3, 9, 10, 28, 30, 31, 36, 42]
	7. Valuing and recognizing SE	• Ensure that students’ input is valued and acted upon • Recognize and celebrate the unique ideas and achievements of SE through meetings, institutional newsletters, and social media • Engaged students showcase their accomplishments and share their activities with the broader community through town halls, social media, bulletins, formal meetings, and face-to-face communications		[1, 8, 9, 28, 31, 37, 39]
**3. Outcomes & achievements**	1. Systems	• Benefits to the whole university/school ° Improve the identification of system issues ° Enhance quality assurance, curriculum development, and faculty/staff development for greater efficiency		[1, 3, 4, 8–10, 28–30, 32, 33, 35, 36, 38–42]
	2. Members	• Benefits to the engaged members ° Recognize the impact of leadership roles on students’ future occupations ° Foster the establishment of beneficial networks among students ° Empower students’ soft skills		[34, 37, 41, 43]

### Models of SE in HPE policy and decision-making

To understand how the models of SE in decision-making bodies are designed and what frameworks assign student representation in HPE governance, we categorized various models based on several identified indicators [9, 30]. In this way, we aim to illustrate how these student-led models cooperate with governance and policy-making bodies [31, 32]. As described in Table 3, we categorized these models into three subthemes: 1. Organization (45.5%), which includes formal and well-structured models established for long-term and sustainable SE; 2. Programs (22.7%), that are informal or less-structured models for usually short-term SE, and 3. Perspective (31.8%), that SE occurs through surveys and interviews.

### Influencing factors of SE in HPE policy and decision-making

Concerning SE in HPE policy and decision-making, several factors play key roles in the establishment, maintenance and fruitfulness of collaborative activities. However, all the factors, including facilitators, challenges, barriers, and suggestions, are categorized into seven subthemes: 1. Structural factors; 2. Environmental factors and institutional culture; 3. Motivational Factors; 4. Members characteristics; 5. Training and mentoring; 6. Members relationship; 7. Valuing and recognizing SE. Details and elements of the subthemes are listed in Table 3.

### Outcomes and achievements of SE in HPE policy and decision-making

The outcomes of engaging students in HPE policy and decision-making activities are related to: 1. Systems: The whole university and school can benefit from SE, e.g., troubleshooting, promoting rules and policies, developing curriculum and promoting staff/faculty; 2. Members: individuals directly involved in SE models and processes attain a systematic and leadership perspective, empower their soft skills, develop valuable networks, and are more aware of making conscious decisions for their future careers (Table 3). Meanwhile, the most prominent achievements of different SE models are incorporating student leaders in educational decision-making to a) better identify system issues and b) improve curriculum development [1, 4, 8, 9, 29, 30, 32, 33, 35, 39–41].

### The interconnection of influencing factors in SE in HPE policy and decision-making

**Fig. 2:**
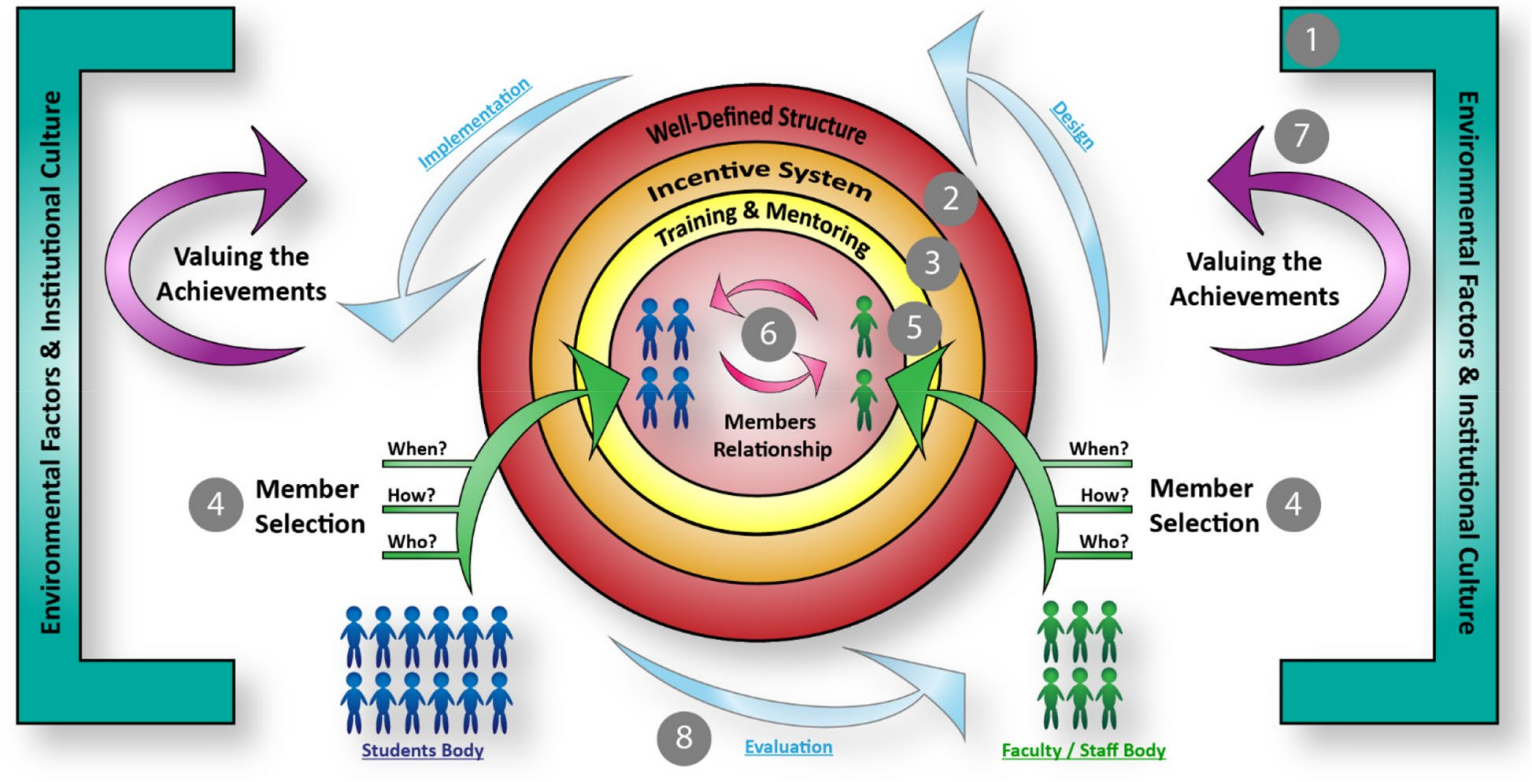
In this picture, it is briefly shown that in order to create an efficient SE model, what steps should be taken and what factors influence it: Step 1,2. According to environmental factors and institutional culture, a well-defined structure is designed. Step 3. Based on motivational factors, an incentive system is created inside the structure. Step 4. Members are selected from student body and faculty/staff body, regarding to three questions Who should involve? How is the process of selection? When the structure call for membership? Step 5. Selected members enter training and mentoring plans. Step 6. The members establish, maintain and improve a high-quality relationship to collaborate on policy and decision-making activities. Step 7. The outcomes and achievements of this collaboration then recognized and valued throughout the institution; as a result, the environment and its culture gradually shift to support the SE. Step 8. Dynamically, the whole process undergoes regular evaluation and correction

By the relational content analysis of the influencing factors in SE in HPE policy and decision-making, we investigated similarities among studies that describe how the dynamic of these factors acts in the real world. Consequently, the authors developed a conceptual model (Fig. 2) presenting 8 steps through which universities can create an efficient and sustainable SE model. This is a practical and integrated presentation of influencing factors listed in Table 3.

## Discussion

According to the literature review, we identified that engaging students in HPE policy and decision-making can have a significant impact on the development of both universities and students. Such collaboration is often implemented through specific SE models, which are influenced by a variety of factors that interact with each other to create these models.

Our research shows that most (81.8%) models of SE have focused on *curriculum development*. This represents the educational universities’ need for student participation to revise, evaluate and improve the curriculum. On the other hand, there have not been enough studies or significant participation in other areas, i.e., planning for the educational program, quality assurance, and faculty/staff development. Thus, future research is needed to investigate the role and impact of SE in the mentioned areas.

### Terms and definitions of SE in HPE policy and decision-making

Based on our research questions, we found several terms and their various definitions in the literature pertaining to SE in policy and decision-making, shown in Table 2. We encountered an overlapping set of vocabularies pointing to student engagement. While these terms could be defined and interpreted differently (e.g., as a spectrum for the level of engagement), the most frequent and exhaustive term is still “Student engagement” [3, 5, 10, 19, 28–30].

Through the data analysis, we found SE models in HPE policy and decision-making and classified them into three categories based on their formality, structuredness, and level of student involvement: *organizations*, *programs*, and *perspectives*. In addition, we elicited seven factors that need to be considered throughout the SE process, especially in a highly structured model. We also determined the outcomes and achievements of SE, which could impact both *members* and *systems*. Besides, we discovered how influencing factors are interconnected to create an effective SE model.

### Models of SE in HPE policy and decision-making

Regarding our findings, SE models place students at the highest level of engagement, and students have a more objective impact on processes and decisions. Our findings reveal that engaging students in HPE policy and decision-making needs to establish formal or informal structures (Organizations and programs) or use the objective tools (perspective) that align with our second research question. If SE models are applied correctly and practically, they will lead to better identification of the educational system issues and the development of the health professions educational curriculum.

### Influencing factors of SE in HPE policy and decision-making

Additionally, some articles have recommended changes to the management framework to shift universities towards systematic engagement of student leaders, rather than just defining SE models [5]. In response to our third research question, we identified several common driving factors related to SE models, which were also found in our findings. These factors include peer mentoring, establishing supportive structures and culture, and optimizing feedback and communication [5, 11, 26].

According to our findings, a handful of studies made payments to support students financially to appreciate the collaborative activities of the students. It is worth mentioning that the students did not consider these receipts as their most important motivation for student participation, and they aimed to promote and improve the educational situation [3, 30]. Based on our review, most of the SE models represented in studies did not prioritize the compensation of student activities with financial rewards in their partnership structure, and the effectiveness of financial motivation requires more study [3, 4, 30].

### Outcomes and achievements of SE in HPE policy and decision-making

Our research aimed to explore the characteristics, functions, and structures of SE models that can lead to a positive outcome from SE. Studies have shown SE in policy and decision-making has potential benefits affecting both the whole university as a system and students as members [1, 4, 8, 29, 30, 33, 35, 39–41]. While other studies have investigated methods for measuring SE and its outcomes, we suggest that future researchers focus on analyzing the outcomes of SE models using these measurement methods [26].

### The interconnection of influencing factors in SE in HPE policy and decision-making

Through relational content analysis of influencing factors, we realized that constructing practical SE models requires a series of steps. Our conceptual model (Fig. 2) suggests that at the beginning of creating SE models, health sciences universities should determine their educational gaps and identify their institutional culture; So that the tasks and roles of students should be well-defined and concordant with these gaps and their environmental features. However, universities should identify their potential and adopt a conciliatory attitude to establish a strong cohesion between SE models and the academic system and create an atmosphere of accepting students’ feedback (Steps 1 and 2) [1, 10, 28, 37]. Next, our research shows that students’ motivations and personality traits, such as leadership, critical thinking, etc. should be assessed before entering into SE models, to arrange the students with their roles properly (Step 3) [3, 10, 28, 30]. Literature indicates that a transparent and disciplined member selection (students and faculty/staff) is a remarked characteristic of SE models (Step 4) [3, 10, 28, 37]. Furthermore, the mentorship and coaching programs before the encounter with the real responsibilities can provide sufficient orientation for the junior members (Step 5) [3, 8, 10, 28, 31, 36, 39]. Finally, creating a safe environment where open dialogues and reciprocal communications flow among the members and students’ views are considered valuable and taken into consideration, makes the SE models more sustainable and efficient (Steps 6 and 7) [3, 10, 28, 30, 31, 37, 39]. Regularly, members’ performance and the SE processes should be evaluated and monitored, to clarify the outcomes of these models (Step 8) [3, 8].

### Limitations and further studies

Our study captures that most of the research papers in this field have been published on SE activities in the Western world; therefore, the extracted themes are based on these studies. Further studies are needed for these cases in other regions of the world, and they may differ to some extent. Considering the themes extracted from the surveys and interviews with relevant people and that most of the studies were qualitative, each strategy needs to be studied in-depth in terms of operational details and effectiveness. The current study has merely included undergraduate medicine, pharmacy, veterinary, and nursing disciplines. Thus, further investigation of other healthcare professions is recommended to derive SE models, outcomes and influencing factors in policy and decision-making activities more comprehensively. This review excluded heterogeneous publications such as perspective articles, opinion pieces and innovations. Hence, we have not considered some studies that could be important for understanding an emerging research space. Moreover, our data collection goes back to November 2022. Thus, some relevant studies might have been published after this date, and we have not included these probable new articles.

## Conclusion

To conclude, the implementation of SE in policy and decision-making is crucial for the advancement of HPE. The models of SE primarily involve organization, programs, and perspective, with universities mainly utilizing SE for curriculum development and quality assurance. Developing SE models not only helps identify issues and generate solutions but also enhances faculty/staff development, leadership roles, and student networking. We categorized the influencing factors of SE in HPE policy and decision-making into seven subthemes: 1. Structural factors; 2. Environmental factors and institutional culture; 3. Motivational Factors; 4. Members characteristics; 5. Training and mentoring; 6. Members relationship; 7. Valuing and recognizing SE. Based on the data analysis of these factors, we proposed a conceptual model that helps HPE universities in practice to develop robust SE models. However, to effectively engage students, universities should adopt SE models that align with their goals and organizational capabilities. We suggest further studies to investigate the impact of SE in policy and decision-making in depth and more objectively.

### Supplementary Information

**Supplementary file 1.**

**Supplementary file 2.**

**Supplementary file 3.**

## Data Availability

The content of the thematic analysis of qualitative data in this study is available from the corresponding author upon reasonable request.

## Abbreviations

SE: Student engagement
HPE: Health professions education
PRISMA-ScR: Systematic Reviews and Meta-Analyses extension for Scoping Reviews

## Footnotes

1 ASPIRE Student Engagement: Criteria and Guidelines for Submitters 2023
