# Supplementary material for: Mapping student engagement in health professions education policy and decision-making: a scoping review
Source: BMC Med Educ. 2024 Mar 22;24:325. doi: 10.1186/s12909-024-05283-8 (PMC10960467; doi:10.1186/s12909-024-05283-8)
Supplement: Supplementary file 1 [file 12909_2024_5283_MOESM1_ESM.docx]

## **Appendix S1: Search Strings**

| **Category** | **Keyword** |
| --- | --- |
| Student engagement | ("student engagement" OR "student partnership" OR "learner engagement" OR “student involvement” OR ”student participation” OR “student contribution” OR "students engagement" OR "students partnership" OR "learners engagement" OR “students involvement” OR ” students participation” OR “students contribution” OR “student–faculty engagement”) AND (govern* OR “policy making” OR “decision making” OR “management” OR “leadership” OR “faculty recruitment” OR “faculty retention” OR “faculty promotion” OR “strategic planning” OR “curriculum development” OR “quality assurance”) |
| Higher education | Universit*  ‘High* education’  College  School  Institutions  faculty  education |

| **Search String (LIMIT: 1999/01/01 – 2022/11/12)** | **Database** | **Results** |
| --- | --- | --- |
| ("student engagement"[TIAB] OR "student partnership"[TIAB] OR "learner engagement"[TIAB] OR “student involvement”[TIAB] OR” student participation”[TIAB] OR “student contribution”[TIAB] OR “students engagement”[TIAB] OR “students partnership”[TIAB] OR “learners engagement”[TIAB] OR “students involvement”[TIAB] OR “students participation”[TIAB] OR “students contribution”[TIAB] OR “student–faculty engagement”[TIAB]) AND (Govern*[TIAB] OR “policy making”[TIAB] OR “decision making”[TIAB] OR “management”[TIAB] OR “leadership”[TIAB] OR “faculty recruitment”[TIAB] OR “faculty retention”[TIAB] OR “faculty promotion”[TIAB] OR “strategic planning”[TIAB] OR “curriculum development”[TIAB] OR “quality assurance”[TIAB]) AND (Universit*[TIAB] OR “High* education”[TIAB] OR “College”[TIAB] OR “School”[TIAB] OR “Institutions”[TIAB] OR “faculty”[TIAB] OR “education”[TIAB]) | PubMed | 392 |
| TS=("student engagement" OR "student partnership" OR "learner engagement" OR “student involvement” OR “student participation” OR “student contribution” OR "students engagement" OR "students partnership" OR "learners engagement" OR “students involvement” OR ” students participation” OR “students contribution” OR “student–faculty engagement”) AND TS=(Govern* OR “policy making” OR “decision making” OR “management” OR “leadership” OR “faculty recruitment” OR “faculty retention” OR “faculty promotion” OR “strategic planning” OR “curriculum development” OR “quality assurance”) AND TS=(Universit* OR “High* education” OR “College” OR “School” OR “Institutions” OR “faculty” OR “education”) | WOS | 2627 |
| TITLE-ABS-KEY ("student engagement" OR "student partnership" OR "learner engagement" OR “student involvement” OR “student participation” OR “student contribution” OR "students engagement" OR "students partnership" OR "learners engagement" OR “students involvement” OR ” students participation” OR “students contribution” OR “student–faculty engagement”) AND TITLE-ABS-KEY (Govern* OR “policy making” OR “decision making” OR “management” OR “leadership” OR “faculty recruitment” OR “faculty retention” OR “faculty promotion” OR “strategic planning” OR “curriculum development” OR “quality assurance”) AND TITLE-ABS-KEY (Universit* OR “High* education” OR “College” OR “School” OR “Institutions” OR “faculty” OR “education”) | Scopus | 3780 |
| ti,ab("student engagement" OR "student partnership" OR "learner engagement" OR “student involvement” OR “student participation” OR “student contribution” OR "students engagement" OR "students partnership" OR "learners engagement" OR “students involvement” OR ” students participation” OR “students contribution” OR “student–faculty engagement”) AND ti,ab(Govern* OR “policy making” OR “decision making” OR “management” OR “leadership” OR “faculty recruitment” OR “faculty retention” OR “faculty promotion” OR “strategic planning” OR “curriculum development” OR “quality assurance”) AND ti,ab(Universit* OR “High* education” OR “College” OR “School” OR “Institutions” OR “faculty” OR “education”) | ProQuest | 582 |

Databases: PubMed, WOS, Scopus, ProQuest, ERIC

Duplicate records: 3069
