# Supplementary material for: Mapping student engagement in health professions education policy and decision-making: a scoping review
Source: BMC Med Educ. 2024 Mar 22;24:325. doi: 10.1186/s12909-024-05283-8 (PMC10960467; doi:10.1186/s12909-024-05283-8)
Supplement: Supplementary file 2 [file 12909_2024_5283_MOESM2_ESM.docx]

**Appendix S2:**

**List of hand-searched medical education journals for the scoping review**

| **Journals** |
| --- |
| Academic Medicine |
| Medical Teacher |
| Medical Education |
| BMC Medical Education |
| Journal of Graduate Medical Education |
| Advances in Medical Education and Practice |
| Medical Education Online |
