# Supplementary material for: Mapping student engagement in health professions education policy and decision-making: a scoping review
Source: BMC Med Educ. 2024 Mar 22;24:325. doi: 10.1186/s12909-024-05283-8 (PMC10960467; doi:10.1186/s12909-024-05283-8)
Supplement: Supplementary file 3 [file 12909_2024_5283_MOESM3_ESM.docx]

| **Characteristics** | **Inclusion** | **Exclusion** |
| --- | --- | --- |
| **Population** | Being part of the undergraduate HPE disciplines (medicine, pharmacy, veterinary, nursing majors) | Students, staff, and faculty of postgraduate HPE or undergraduate non-HPE |
| **Context** | Health sciences universities | Other higher education universities |
| **Concept** | The focus of the research is on SE in HPE policy and decision-making activities in universities, such as SE forms, their outcomes, and influencing factors. | Studies involving students merely in clinical decision-making, teaching and learning policies and just in curriculum evaluation |
| **Types of evidence source** | Peer-reviewed articles published or on-press in English | - Non-English papers  - Articles with no existing full texts  - Conference abstracts, letters and editorials |

**Appendix S3:**

**Detailed inclusion and exclusion criteria**
